# Clozapine to treat aggression and agitation in advanced dementia

**DOI:** 10.1192/j.eurpsy.2024.492

**Published:** 2024-08-27

**Authors:** A. E. Michael, N. Michael, A. Erfurth, M. Kujovic

**Affiliations:** ^1^Johannes Wesling Klinikum Minden, Minden; ^2^Krankenhaus Elbroich, Düsseldorf, Germany; ^3^1st Department of Psychiatry and Psychotherapeutic Medicine, Klinik Hietzing, Vienna, Austria; ^4^LVR-Klinikum , Düsseldorf, Germany

## Abstract

**Introduction:**

Agitation and aggression are a serious problem in clinical psychiatry, especially in multimorbid patients of advanced age, including those with dementia.

**Objectives:**

We wanted to investigate to what extent clozapine could be an option in the treatment of selected refractory patients.

**Methods:**

A retrospective study included patients with a diagnosis of dementia who were treated with clozapine in a specialist geriatric psychiatry unit between August 2018 and February 2022, and medical records were systematically reviewed. The Clinical Global Impressions Scale was used for the assessment of improvement and the Pittsburgh Agitation Scale for the assessment of symptom reduction. In addition, there was detailed documentation of side effects and clinical features.

**Results:**

A total of 31 patients with a median age of 82 years were identified.

**Conclusions:**

In conclusion, clozapine was effective and well tolerated in 23 patients. This suggests that low-dose clozapine may help alleviate the suffering of difficult-to-treat multimorbid patients with advanced dementia and their carers. However, adverse effects, particularly in patients with cardiovascular and pulmonary impairment, should be carefully monitored.

**Disclosure of Interest:**

None Declared

